# All That Is Wide Is Not Ventricular Tachycardia: A Case Highlighting Supraventricular Tachycardia as an Alternative in Stable Wide Complex Tachycardia With Reversible Cardiomyopathy

**DOI:** 10.7759/cureus.104485

**Published:** 2026-03-01

**Authors:** Admire Hlupeni, Rabbia Haider, Jonas Cooper

**Affiliations:** 1 Internal Medicine, St. Luke's Hospital, Chesterfield, USA; 2 College of Health Sciences, Midlands State University, Gweru, ZWE; 3 Cardiology, St. Luke's Hospital, Chesterfield, USA

**Keywords:** cardiac mri, electrophysiology, familial svt, holiday heart, non-ischemic cardiomyopathy, supraventricular tachycardia (svt), svt with aberrancy, tachycardia-induced cardiomyopathy (tic), ventricular tachycardia (vt), wide complex tachycardia (wct)

## Abstract

Wide complex tachycardia (WCT) is often treated as ventricular tachycardia (VT). However, it can also be supraventricular tachycardia (SVT) with aberrant conduction, which is rarely life-threatening. Accurately differentiating VT from SVT with aberrancy guides appropriate therapy. We present a case that highlights the value of structured electrocardiogram (ECG) analysis in hemodynamically stable WCT and demonstrates reversible alcohol-related SVT-induced cardiomyopathy in a patient with familial susceptibility. A 51-year-old previously healthy woman presented to urgent care with four hours of palpitations and sharp substernal chest pain radiating to the right neck, accompanied by anxiety, nausea, and mild dyspnea. She had consumed alcohol heavily two days earlier and was hemodynamically stable. ECG showed a regular borderline WCT (QRS 114 milliseconds) at 192 beats per minute, flagged by machine interpretation as possible acute MI, prompting urgent transfer to the emergency department (ED). En route, she received IV amiodarone with resolution of symptoms. In the ED, repeat ECG revealed sinus WCT (QRS 121 milliseconds, at 105 beats per minute) with a left bundle branch block. Troponins and electrolytes were normal. Echocardiogram demonstrated a left ventricular ejection fraction (LVEF) of 22%. Invasive coronary angiography showed no obstructive disease. Cardiac MRI revealed no late gadolinium enhancement. She was started on full guideline-directed medical therapy (GDMT). Further history revealed prior vagal-responsive palpitations and a child with SVT requiring ablation. She was discharged with a 14-day ambulatory monitor showing SVT burden <0.01%. At the three-month follow-up, her LVEF had normalized to 56%. Carvedilol was discontinued due to bradycardia, while other GDMT was continued. This case highlights the value of structured ECG analysis in hemodynamically stable WCT, where careful review, using tools like the Brugada criteria, can distinguish SVT with aberrancy from VT and avoid inappropriate exposure to amiodarone. Although empiric treatment for presumed VT is reasonable when uncertainty exists, clinical stability often allows time for systematic ECG assessment to prevent overtreatment. The patient’s complete recovery demonstrates the reversibility of TIC and reinforces the importance of continuing GDMT even after LVEF normalization to reduce relapse risk.

## Introduction

Wide complex tachycardia (WCT) presents a significant diagnostic challenge, especially when accompanied by symptoms suggestive of myocardial infarction (MI). WCT refers to a tachyarrhythmia with a QRS duration ≥120 ms, which may reflect either ventricular origin or supraventricular rhythm conducted via an abnormal intraventricular conduction pathway (aberrancy). Because ventricular tachycardia (VT) accounts for most WCT in adults, clinicians often default to treating WCT as VT [1,2]. However, supraventricular tachycardia (SVT) with aberrancy remains an important alternative, and distinguishing the two is essential, as their management strategies differ markedly: VT generally requires urgent antiarrhythmics or cardioversion, whereas SVT with aberrancy may respond safely to AV nodal blockade [1,3]. Misclassification may therefore delay appropriate therapy or expose patients to unnecessary and potentially toxic treatments.

We report a case initially treated empirically as VT but ultimately determined to be SVT with aberrant conduction. This case highlights how hemodynamic stability provides a window for structured electrocardiogram (ECG) analysis, using tools such as the Brugada criteria [4], to refine rhythm diagnosis and avoid unnecessary broad-spectrum antiarrhythmics like amiodarone. It also underscores the value of integrating clinical history, imaging, and ambulatory monitoring when evaluating new-onset cardiomyopathy.

By illustrating the intersection of acute arrhythmia, diagnostic uncertainty, and reversible myocardial dysfunction, this case reinforces the need for diagnostic precision in WCT and supports emerging guidance on the recognition and management of tachycardia-induced cardiomyopathy (TIC).

## Case presentation

A 51-year-old woman presented to an urgent care center with a four-hour history of palpitations and sharp substernal chest pain radiating to the right side of her neck, accompanied by anxiety, nausea, and mild dyspnea that had awakened her from sleep. She had a medical history significant for anxiety, treated with bupropion. She was married, lived with her husband, reported occasional alcohol use, and denied tobacco or illicit drug use. Her family history was notable for congestive heart failure in her father and maternal grandmother. Two days prior to presentation, the patient had engaged in heavy alcohol consumption.

**Figure 1:**
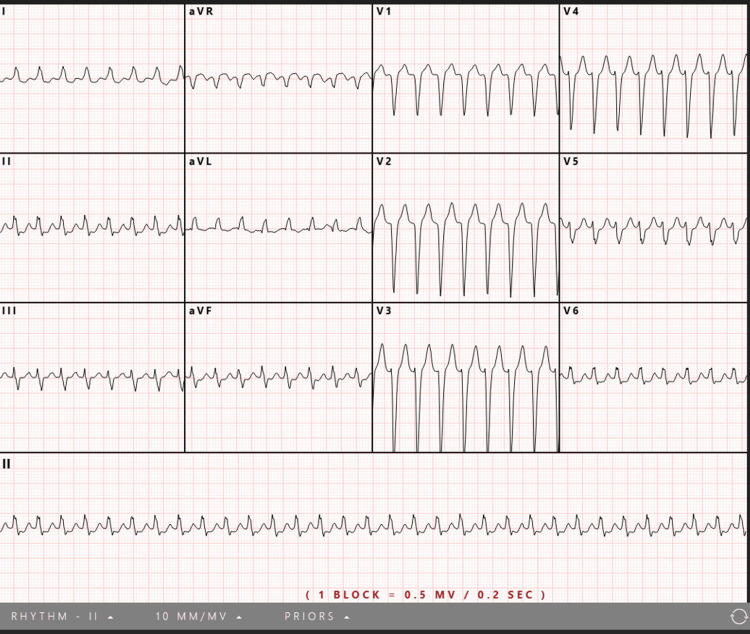
Initial electrocardiogram at urgent care demonstrating a regular tachycardia. The automated analysis reported a ventricular rate of 192 beats per minute, QRS duration of 114 milliseconds, and QT/QTc of 246/344 milliseconds. The computerized interpretation suggested supraventricular tachycardia with possible acute anteroseptal myocardial infarction.

On arrival, she was hemodynamically stable, with blood pressure 113/90 mmHg, respiratory rate 20 breaths per minute, and oxygen saturation 99% on room air. Review of systems was otherwise unremarkable. An ECG demonstrated a regular borderline WCT (QRS 114 milliseconds) at 192 beats per minute, flagged by machine interpretation as possible acute anteroseptal myocardial infarction (Figure 1)

Emergency medical services were contacted within 10 minutes of presentation. She received intravenous fluids and nitroglycerin and was transferred promptly to the emergency department (ED). She had self-administered aspirin 324 mg at home. En route, she received an intravenous amiodarone bolus, resulting in resolution of palpitations and improvement in symptoms.

**Figure 2:**
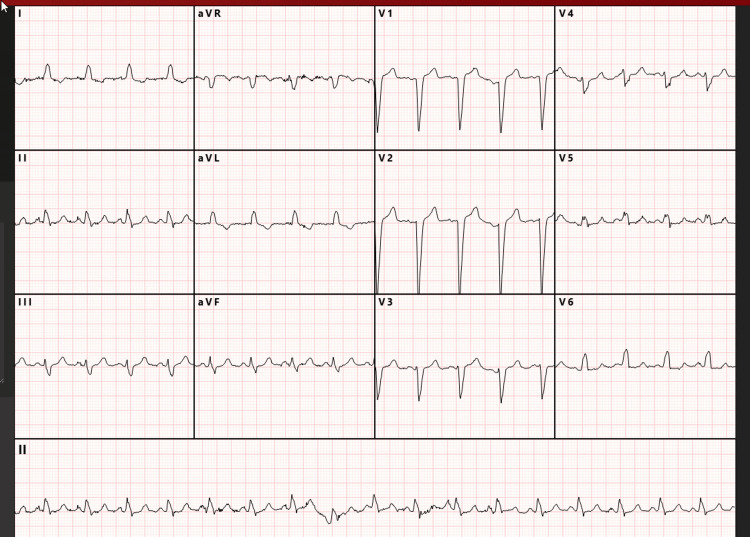
Post-amiodarone electrocardiogram in the emergency department showing sinus tachycardia with left bundle branch block morphology. An electrocardiogram obtained in the emergency department demonstrated a wide-complex tachycardia. Automated analysis reported a ventricular rate of 105 beats per minute, QRS duration of 121 milliseconds, and QT/QTc of 351/412 milliseconds.

In the ED, she was fully alert and oriented. Vital signs were stable: blood pressure 121/90 mmHg, oxygen saturation 99-100%, and respiratory rate 20 breaths per minute. Her palpitations, nausea, and anxiety had resolved. Repeat ECG showed sinus WCT (QRS 121 milliseconds) at 105 beats per minute with a left bundle branch block (LBBB) morphology (Figure 2).

Physical examination and serial laboratory evaluation, including electrolytes and troponins, were unremarkable (Table 1). 

**Table 1:** Initial laboratory results within normal reference ranges. * Serial measurements at two and six hours remained stable at 0.08 ng/mL

Laboratory tests	Reference Ranges	Patient Values
White Blood Count	4.3-10.0 K/µL	6.6
Hemoglobin	13.6-16.5g/dL	11.9
Hematocrit	40-48 %	35.7
Mean Corpuscular Volume	82.0-99.0 fL	94.2
Platelets	140-350 K/ µL	272
Sodium	137-145 mmol/L	138
Potassium	3.4-5.1 mmol/L	4.3
Chloride	98-107 mmol/L	105
Carbon dioxide	22-30 mmol/L	23
Blood urea nitrogen	9-20 mg/dL	10
Creatinine	0.7-1.30 mg/dL	0.9
Glucose	74-106 mg/dL	106
Calcium	8.4-10.2 mg/dL	9.4
Magnesium	1.6-2.6mg/dL	1.8
Troponin I*	<0.12 ng/mL	0.02
N-terminal pro-B-type Natriuretic Peptide	pg/mL	923
Thyroid Stimulating Hormone	0.47-4.68 uIU/mL	4.51
Hemoglobin A1c	<5.6 %	5.0
Total Cholesterol (Fasting)	<=199 mg/dL	168
Direct High Density Lipoprotein	>=41 mg/dL	75
Low Density Lipoprotein (calculated)	<=99	74
Human Chorionic Gonadotropin hormone, Urine Qualitative	-	negative

Transthoracic echocardiography revealed a left ventricular ejection fraction (LVEF) of 22% with global hypokinesis.

**Figure 3:**
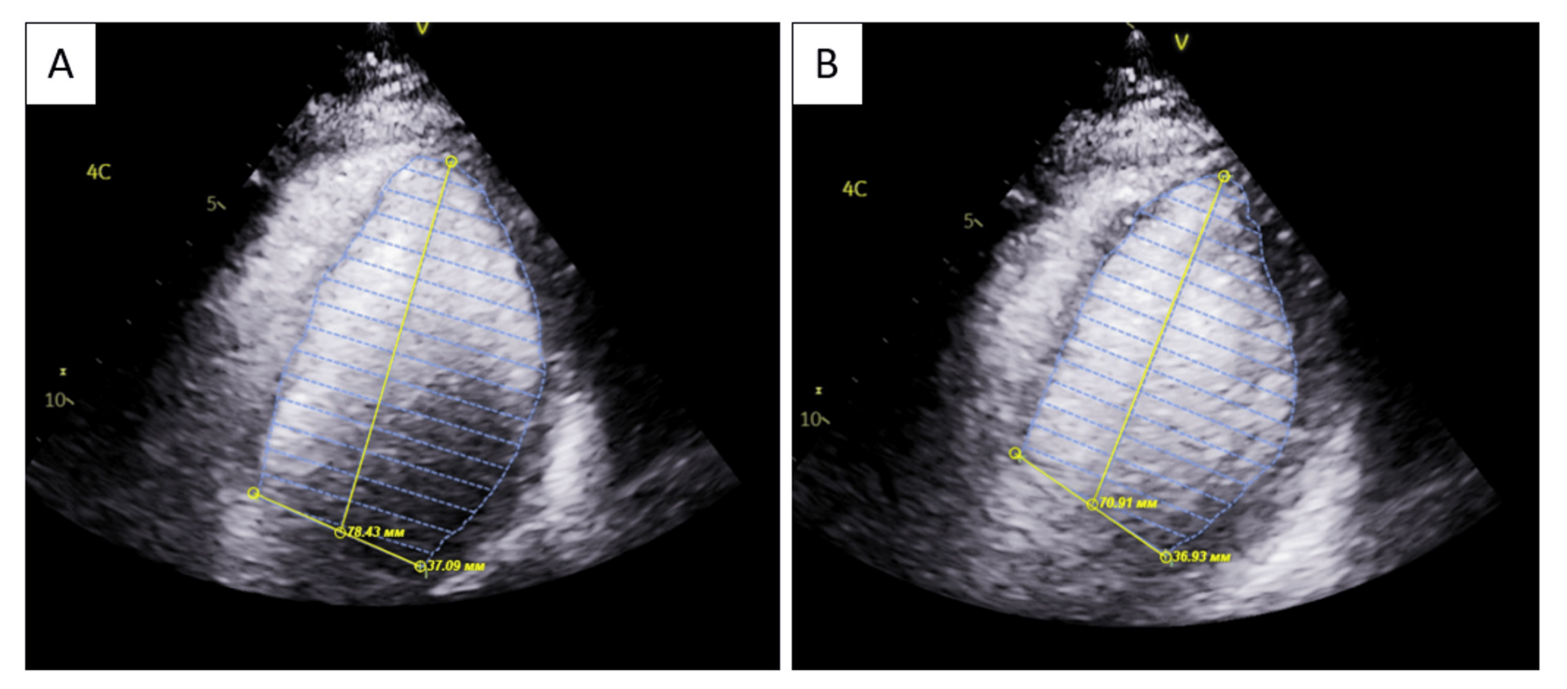
Transthoracic echocardiogram demonstrating reduced left ventricular systolic function. Apical four-chamber view. Panel A shows the left ventricle in end-diastole with increased left ventricular internal diameter in diastole (7.8 cm) and left ventricular end-diastolic volume measured in the apical four-chamber view (105 mL). Panel B shows the end-systolic phase with increased left ventricular internal diameter in systole (7.1 cm) and left ventricular end-systolic volume (85 mL), consistent with reduced systolic function. Linear dimensions and volumetric measurements were derived using different echocardiographic methods, which may account for the apparent discrepancy between diameters and calculated volumes.

Further history revealed a three-year pattern of episodic palpitations that reliably terminated with vagal maneuvers but had never been evaluated. Her child had undergone catheter ablation for SVT at age 11, raising suspicion for a familial arrhythmia predisposition.

The patient was admitted to telemetry for further management. Coronary angiography performed the following day demonstrated no obstructive coronary artery disease. Cardiac MRI (Figure 3) revealed no late gadolinium enhancement, edema, or infiltrative disease, supporting a non-ischemic, potentially reversible cardiomyopathy.

**Figure 4:**
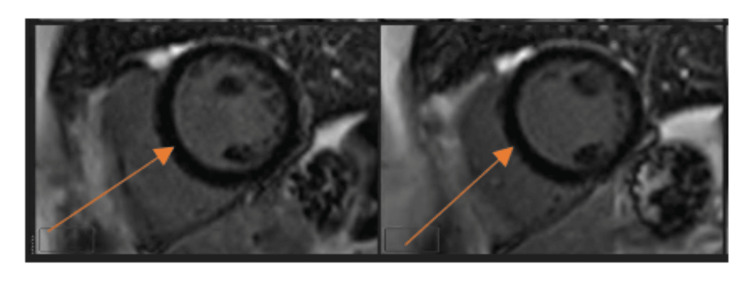
Cardiac magnetic resonance imaging demonstrating normal myocardial tissue characterization. Late gadolinium enhancement images showing no myocardial enhancement (orange arrows), consistent with absence of myocardial fibrosis or scar.

She was started on full guideline-directed medical therapy (GDMT) for heart failure, including sacubitril-valsartan, spironolactone, dapagliflozin, and a beta-blocker. Her heart rate stabilized, and she remained asymptomatic. She was discharged on hospital day 3 with a 14-day ambulatory monitor, which demonstrated a minimal SVT burden (<0.01%).

At the three-month follow-up, her LVEF had normalized to 56%. Carvedilol was discontinued due to bradycardia, while other GDMT was continued in accordance with heart failure management guidelines [5,6].

## Discussion

The initial rhythm presented a diagnostic dilemma: a regular borderline WCT in a hemodynamically stable patient. Although empiric treatment for VT is appropriate when it cannot be excluded, her clinical stability provided an important opportunity for structured ECG analysis. Retrospective application of tools such as the Brugada criteria revealed features supporting SVT with aberrancy, including absence of a dominant R wave in V1, a smooth, unnotched R-wave upstroke, and lack of Q waves in V6 [7,8]. Earlier use of such diagnostic frameworks might have supported the trial of AV nodal blockade and avoided exposure to amiodarone, which carries greater long-term toxicity. This case illustrates the value of deliberate ECG interpretation in stable WCT before committing to empiric therapy.

The patient’s markedly reduced LVEF was ultimately attributed to TIC, a fully reversible cardiomyopathy resulting from sustained or recurrent tachyarrhythmias [9,10]. The absence of coronary artery disease, lack of late gadolinium enhancement on cardiac MRI, global hypokinesis, and fairly rapid normalization of left ventricular function all support TIC as the underlying etiology. Her very low SVT burden on ambulatory monitoring further supported that the ventricular dysfunction resulted from the initial prolonged tachyarrhythmia rather than an ongoing arrhythmia substrate from underlying structural cardiomyopathy [9,10].

Alcohol likely served as the precipitating trigger, consistent with holiday heart syndrome, in a patient with underlying genetic susceptibility. Alcohol-related myocardial stunning was also a potential explanation for this patient's presentation [11,12]. Her history of vagal-responsive palpitations and a child with SVT suggest a possible familial predisposition to reentrant arrhythmias. Recognition of genetic or familial patterns is clinically important, as such patients may have intermittent, trigger-dependent presentations [5,9].

Despite recovery of left ventricular function, GDMT was continued. This adheres to contemporary heart failure guidance, which supports ongoing neurohormonal blockade in reversible cardiomyopathies due to the risk of relapse, especially when the underlying trigger, such as alcohol exposure, may recur [5,6].

In summary, this case highlights two key principles: (i) hemodynamic stability in WCT should prompt structured ECG evaluation using tools such as the Brugada criteria to refine the diagnosis and guide safer initial therapy; and (ii) TIC is fully reversible, but ongoing GDMT and trigger avoidance remain important, particularly in patients with possible genetic predisposition.

## Conclusions

This case highlights the importance of diagnostic precision when evaluating WCT in hemodynamically stable patients. Structured ECG analysis using validated algorithms can improve rhythm classification and support more appropriate initial therapy. It also demonstrates that TIC can present with severe but reversible left ventricular dysfunction and should be considered when imaging reveals no structural disease. Recognition of potential triggers, such as acute alcohol exposure and possible familial susceptibility, is essential for the prevention of recurrence. Even after recovery of ventricular function, continuation of GDMT and close follow-up remain important to reduce relapse risk and support sustained myocardial recovery. Overall, this case reinforces the need for careful rhythm interpretation, integration of multimodality evaluation, and longitudinal management in patients presenting with WCT.
